# Supplementary material for: Whole-limb scaling of muscle mass and force-generating capacity in amniotes
Source: PeerJ. 2021 Nov 29;9:e12574. doi: 10.7717/peerj.12574 (PMC8638577; doi:10.7717/peerj.12574)
Supplement: Supplemental Information 1 [file peerj-09-12574-s001.pdf]

Supplemental Information for

Whole-limb scaling of muscle mass and force-generating capacity in amniotes

Peter J. Bishop[1,2,*], Mark A. Wright[1] and Stephanie E. Pierce[1]

[1]Museum of Comparative Zoology and Department of Organismic and Evolutionary Biology, Harvard University, Cambridge, Massachusetts, USA
[2]Geosciences Program, Queensland Museum, Brisbane, Queensland, Australia

*Correspondence to: pbishop@fas.harvard.edu

Contents:

1. Supplemental text
2. Supplemental figures
3. Supplemental tables
4. Supplemental code

1. Supplemental text

Median size-normalized isometric strength (F_{max}*) v. body mass (comparison 2b)

Qualitatively, results parallel the patterns reported for mean F_{max}* in the main text, but on a finer quantitative scale there are several differences (Fig. S2). In the forelimb, reptiles exhibit negative allometry, driven by the proximal limb, whereas mammals do not significantly depart from isometry overall or in the proximal limb, but exhibit positive allometry in the distal limb. In the hindlimb, both mammals and bipeds exhibit positive allometry at the level of the whole limb, although in mammals this is driven by the proximal limb whereas in bipeds it is driven by the distal limb, which again displays very strong positive allometry. Reptiles only exhibit a significant departure from isometry in the distal hindlimb (negative), and excluding the CFL does not significantly alter scaling patterns (Fig. S6). Analyses of covariance without accounting for phylogeny produce similar results to mean F_{max}* above (Tables S2, S6), indicating that mammals, reptiles and bipeds frequently show different allometric trajectories, especially in terms of slope. Again, differences in intercept were mostly detected for the forelimb, where reptiles exhibit a markedly lower intercept than mammals. Every difference between mammals and reptiles disappears after phylogeny is accounted for using pANCOVA; in contrast, most differences between mammals and bipeds, and reptiles and bipeds, were retained following phylogenetic correction.

Total size-normalized isometric strength v. body mass (comparison 2c)

Qualitatively, results parallel the patterns reported for mean F_{max}*, but on a finer quantitative scale there are a couple of differences (Fig. S3). Both mammals and reptiles ubiquitously do not show significant departure from isometry, and excluding the CFL does not significantly alter the reptile scaling patterns (Fig. S7). Again, bipeds exhibit strong positive allometry throughout the hindlimb, particularly in the distal limb. Analyses of covariance without accounting for phylogeny produce similar results to mean and median F_{max}* (Tables S3, S7), indicating that mammals, reptiles and bipeds frequently show different allometric trajectories in terms of both slope and intercept. Reptiles exhibit a markedly lower intercept than mammals throughout the forelimb, and also exhibit a lower intercept in the distal hindlimb. Almost all differences between mammals and reptiles disappear after phylogeny is accounted for using pANCOVA, whereas most differences between mammals and bipeds, and reptiles and bipeds, were retained following phylogenetic correction.

2. Supplemental figures

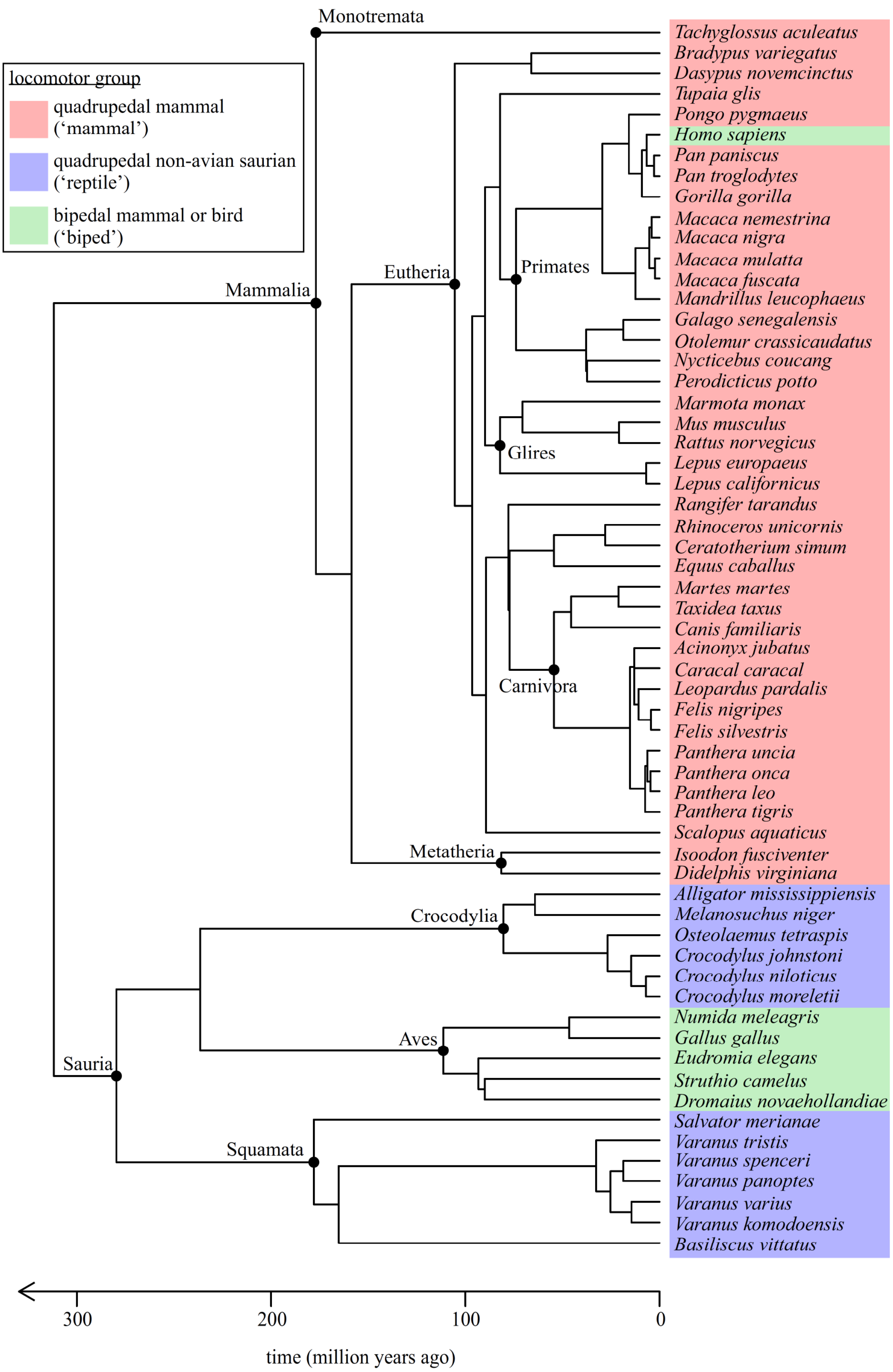

Figure S1. Time-calibrated phylogeny of all taxa analysed in the study, indicating the major locomotor group to which each taxon was assigned for the purpose of statistical analysis. Also labelled are major taxa sampled in the study.

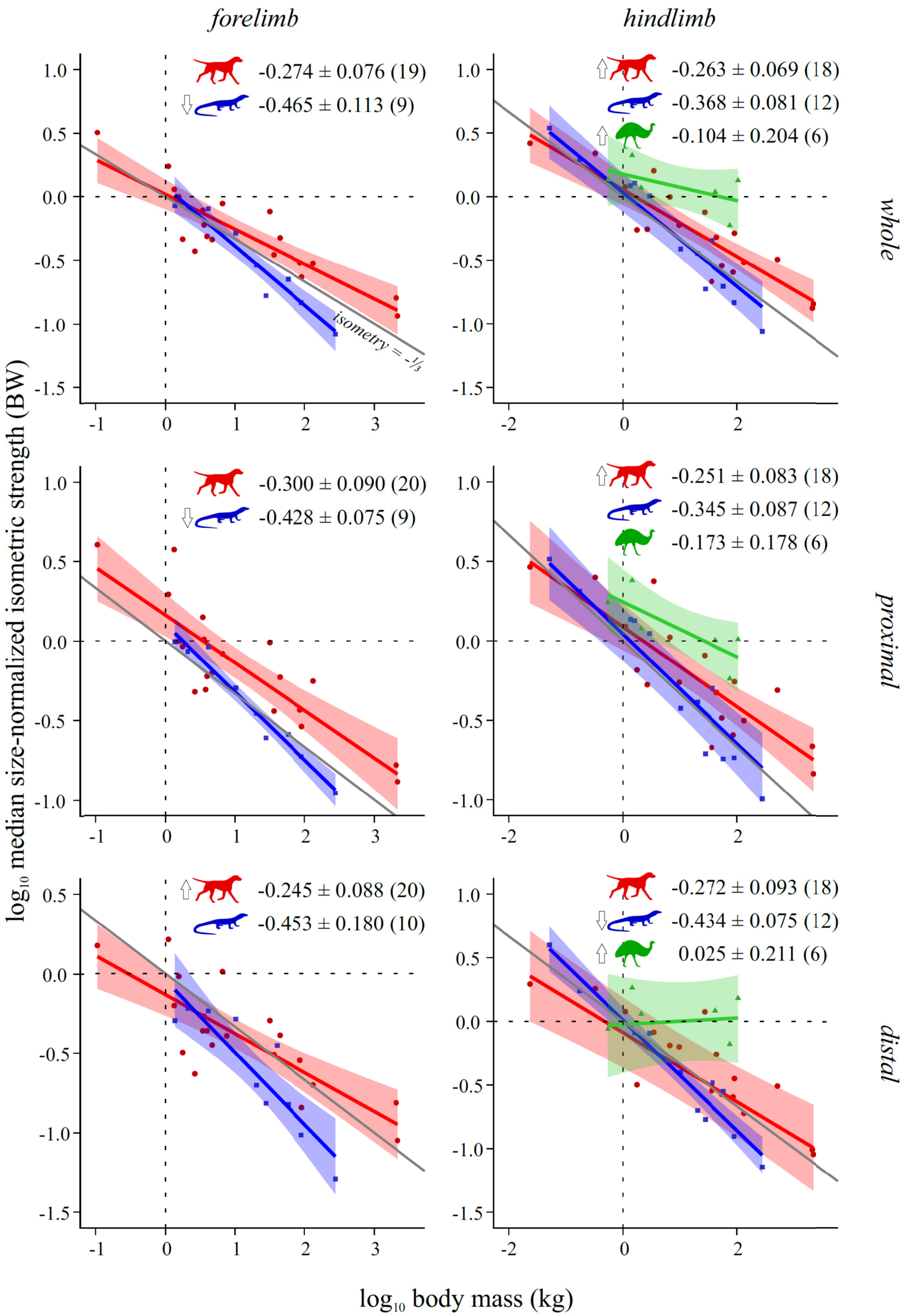

Figure S2. Median size-normalized isometric strength v. body mass across extant terrestrial amniotes. Conventions as in Fig. 2.

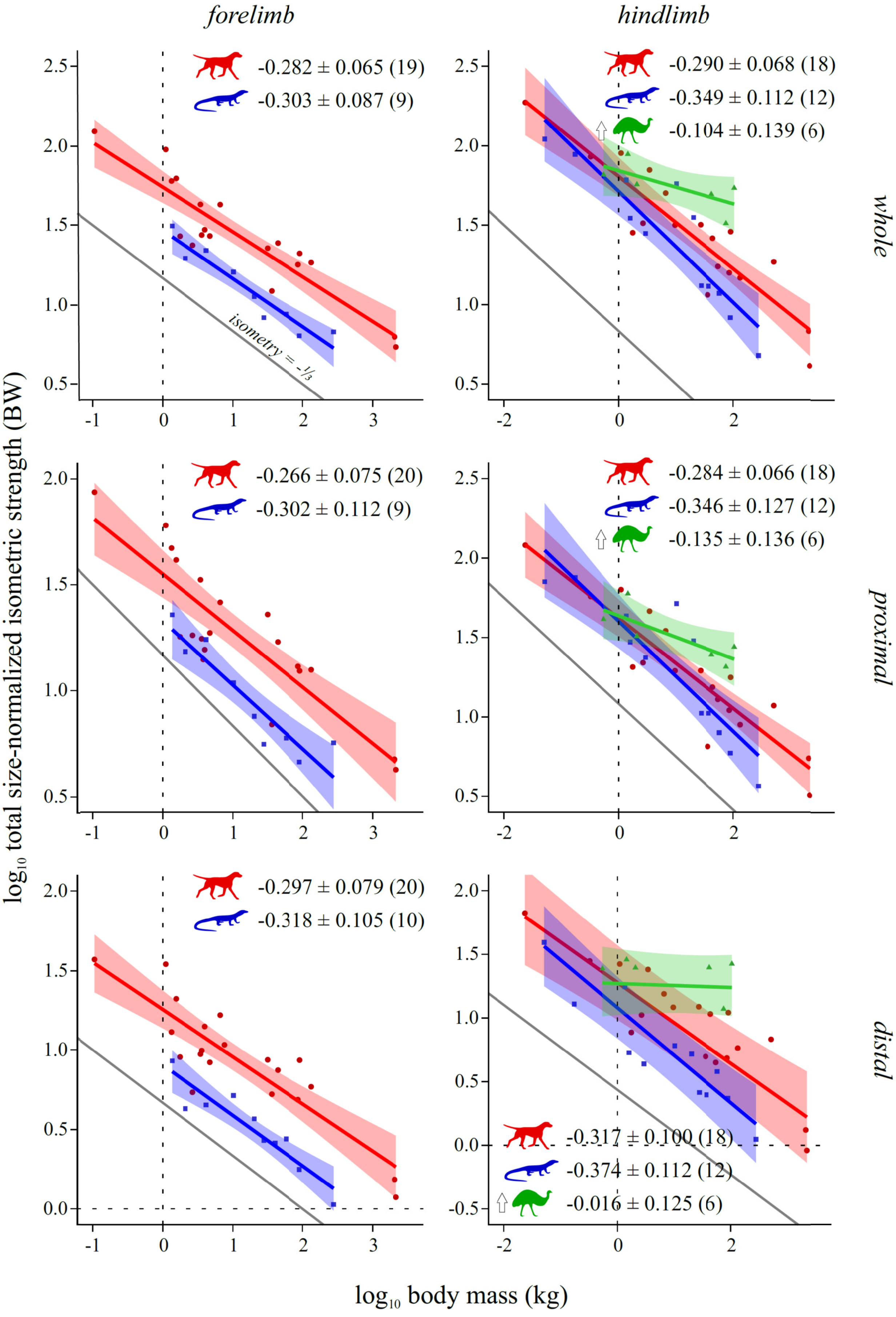

Figure S3. Total size-normalized isometric strength v. body mass across extant terrestrial amniotes. Conventions as in Fig. 2.

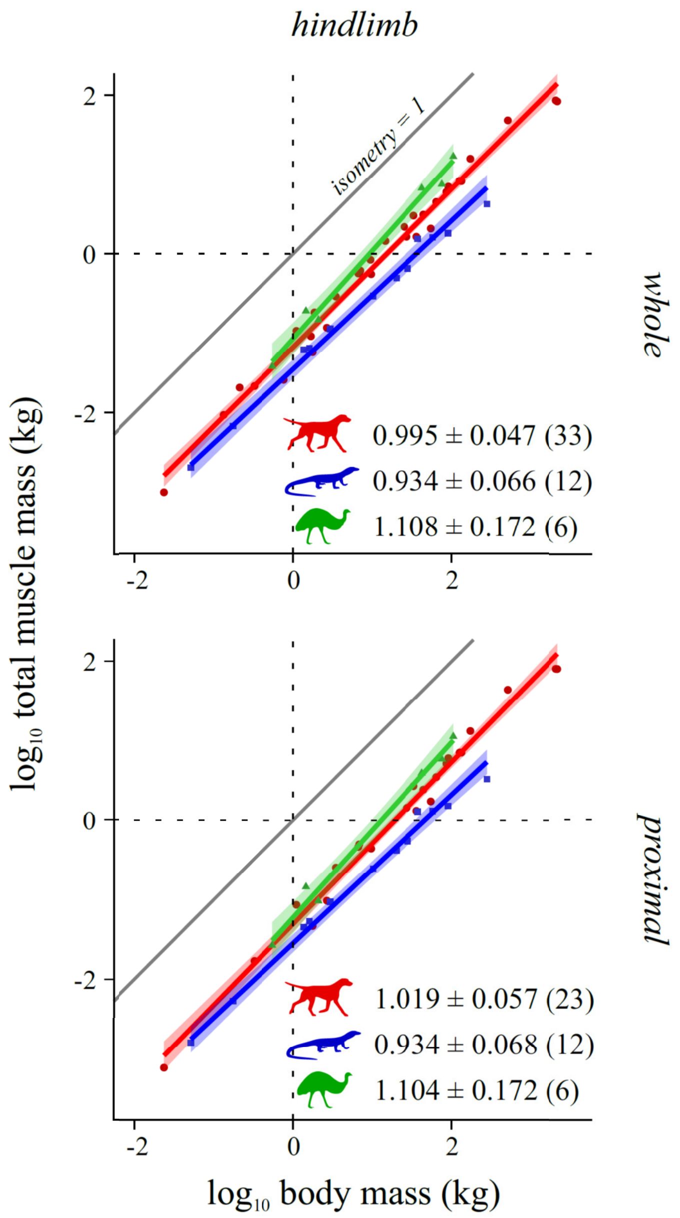

Figure S4. Total hindlimb muscle mass (Σm_{muscle}) v. body mass (m_{body}) across extant terrestrial amniotes, excluding the reptilian caudofemoralis longus. Conventions as in **F**ig. 2.

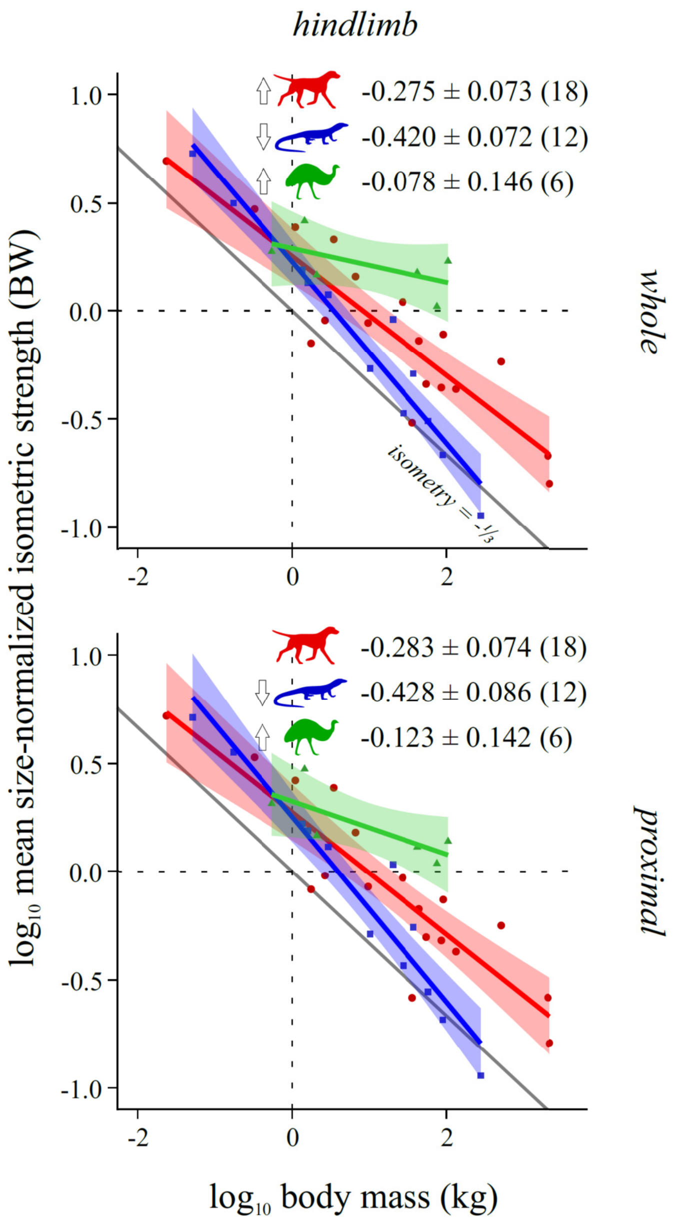

Figure S5. Mean size-normalized isometric strength (F_{max}*) v. body mass (m_{body}) across extant terrestrial amniotes, excluding the reptilian caudofemoralis longus. Conventions as in Fig. 2.

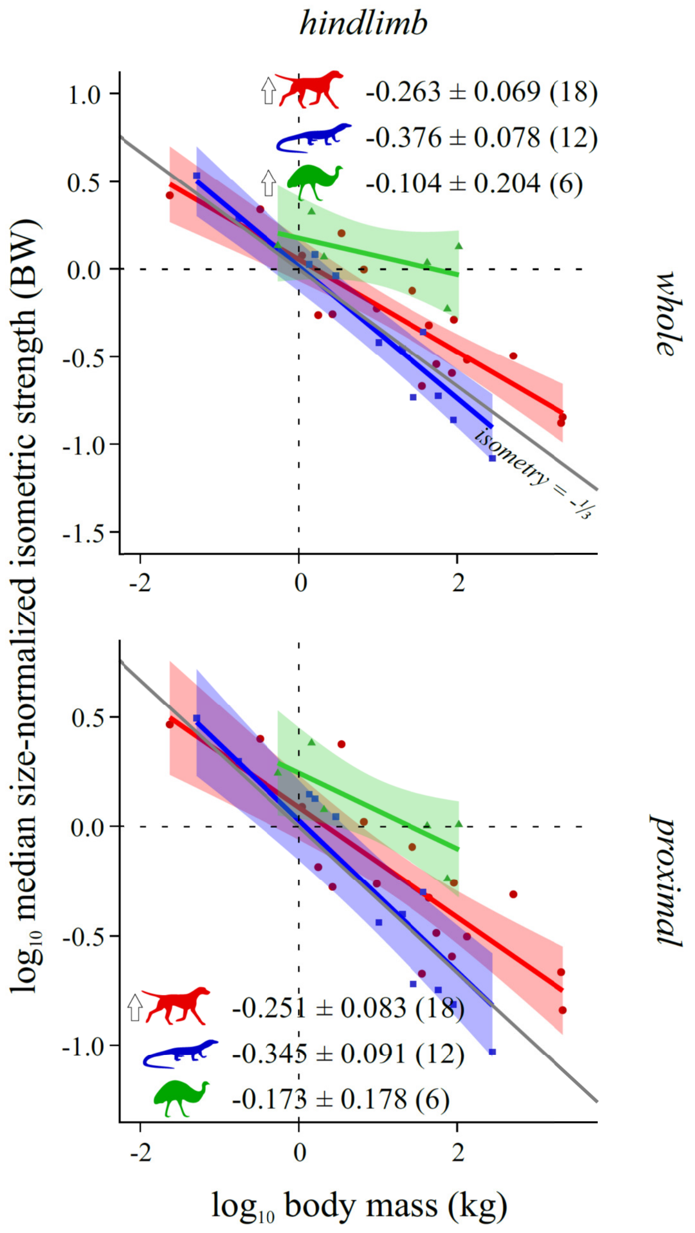

Figure S6. Median size-normalized isometric strength (F_{max}*) v. body mass (m_{body}) across extant terrestrial amniotes, excluding the reptilian caudofemoralis longus. Conventions as in Fig. 2.

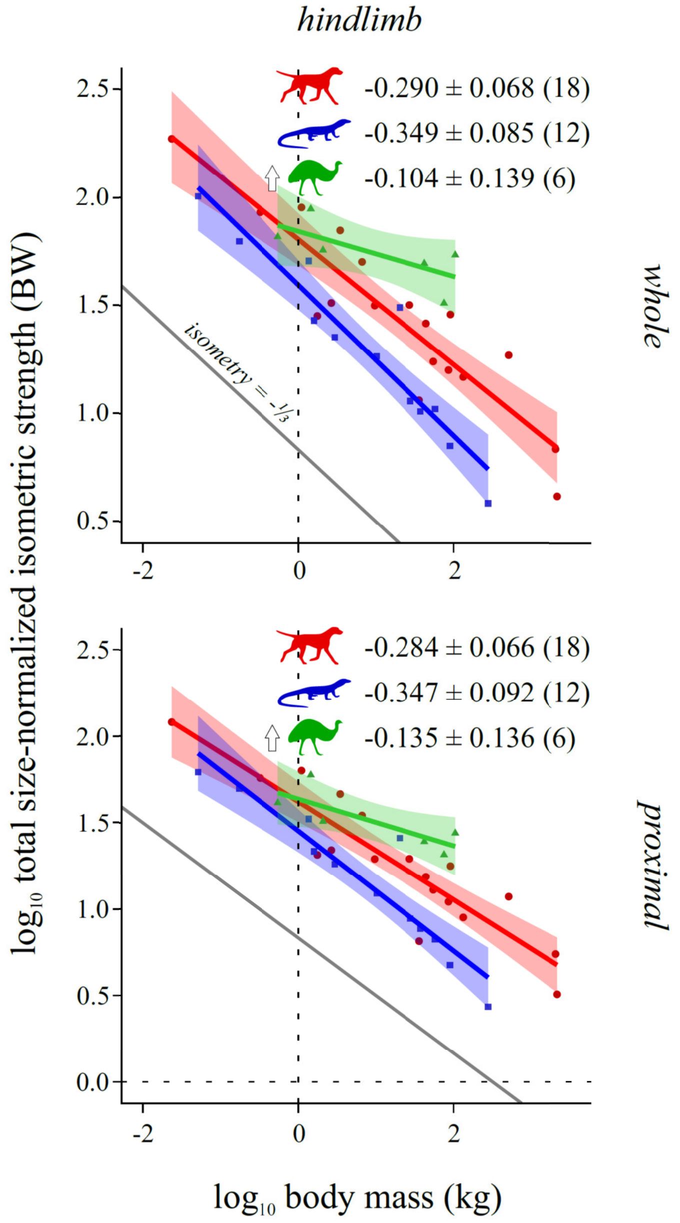

Figure S7. Total size-normalized isometric strength (F_{max}*) v. body mass (m_{body}) across extant terrestrial amniotes, excluding the reptilian caudofemoralis longus. Conventions as in Fig. 2.

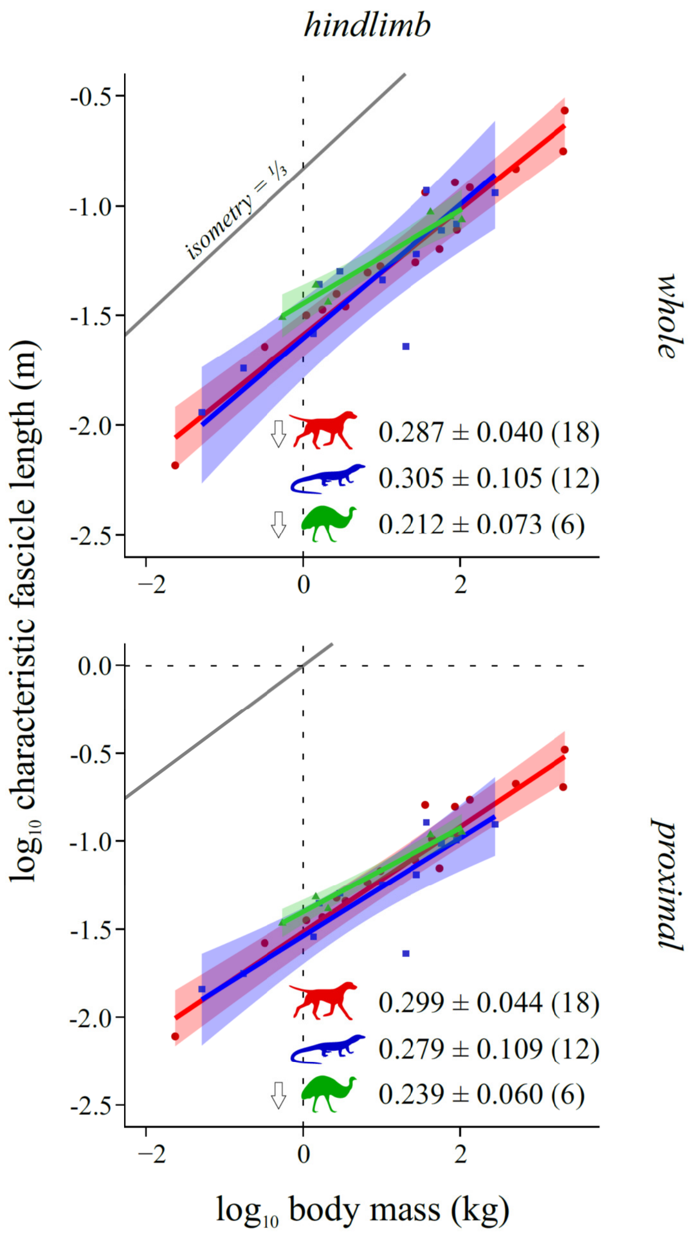

Figure S8. Characteristic fascicle length (L*) v. body mass (m_{body}) across extant terrestrial amniotes, excluding the reptilian caudofemoralis longus. Conventions as in **F**ig. 2.

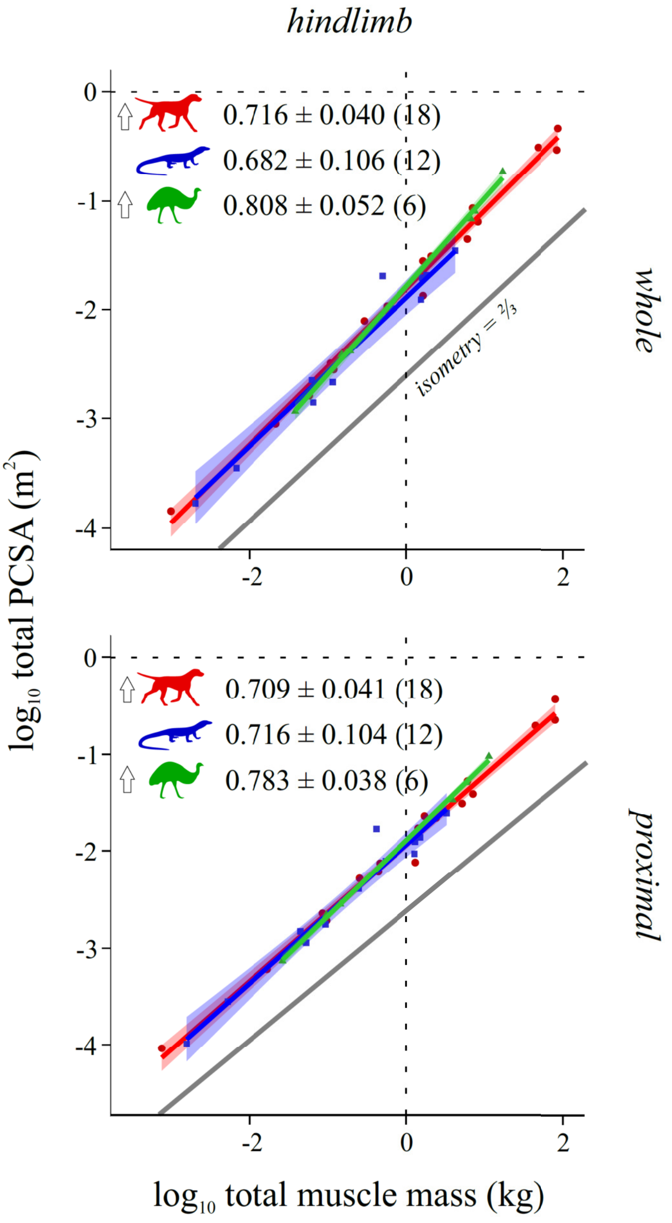

Figure S9. Total physiological cross-sectional area (ΣPCSA) v. total muscle mass ($\Sigma m_{\mathrm{muscle}}$) across extant terrestrial amniotes, excluding the reptilian caudofemoralis longus. Conventions as in Fig. 2.

3. Supplemental tables

Table S1. The raw architectural data used in this study, presented in an Excel worksheet. This file contains four tabs: raw hindlimb data, raw forelimb data, hindlimb data collated into the parameters investigated in the study, and forelimb data collated into the parameters investigated in the study. The raw data for the opossum (*Didelphis virginiana*) and tegu lizard (*Salvator merianae*) dissected here are reported as mean ± one standard deviation for fascicle length and pennation angle.

Table S2. Results of comparisons between each major group via pANCOVA and ANCOVA, for the analysis of median F_{max}* v. m_{body}. Each pairwise comparison was tested for differences in slope (S), intercept (I) and slope and intercept (S + I). Results for analyses without controlling for phylogeny are also presented (ANCOVA, †); significant results are in boldface; *df* = degrees of freedom.

**anatomical region**		**test**	**mammals v. reptiles**					**mammals v. bipeds**					**reptiles v. bipeds**				
			df	***F***	***P***	***F*†**	***P*†**	***df***	***F***	***P***	***F*†**	***P*†**	***df***	***F***	***P***	***F*†**	***P*†**
		S	2,25	4.94	**0.036**	13.428	**0.001**										
	**whole**	I	2,25	0.967	0.335	6.115	**0.021**										
		S + I	2,24	2.492	0.104	6.65	**0.005**										
		S	2,26	3.448	0.075	10.782	**0.003**										
**forelimb**	**proximal**	I	2,26	1.997	0.17	8.244	**0.008**										
		S + I	2,25	1.714	0.201	5.321	**0.012**										
		S	2,27	3.709	0.065	9.487	**0.005**										
	**distal**	I	2,27	0.747	0.395	4.319	**0.047**										
		S + I	2,26	1.919	0.167	4.855	**0.016**										
		S	2,27	2.492	0.126	16.152	**<0.001**	2,21	7.619	**0.012**	14.396	**0.001**	2,15	27.093	**<0.001**	50.166	**<0.001**
	**whole**	I	2,27	0.133	0.718	4.335	0.047	2,21	2.738	0.113	10.571	**0.004**	2,15	4.879	**0.043**	14.79	**0.002**
		S + I	2,26	1.2	0.317	7.815	**0.002**	2,20	3.651	**0.045**	7.763	**0.003**	2,14	13.277	**0.001**	25.83	**<0.001**
		S	2,27	1.704	0.203	12.452	**0.002**	2,21	2.262	0.148	5.501	**0.029**	2,15	13.515	**0.002**	32.281	**<0.001**
**hindlimb**	**proximal**	I	2,27	0.252	0.62	4.133	0.052	2,21	1.229	0.28	6.399	**0.02**	2,15	6.123	**0.026**	17.189	**0.001**
		S + I	2,26	0.847	0.44	5.995	**0.007**	2,20	1.083	0.358	3.478	0.051	2,14	7.668	**0.006**	18.881	**<0.001**
		S	2,27	2.3	0.141	14.534	**0.001**	2,21	13.104	**0.002**	19.487	**<0.001**	2,15	37.946	**<0.001**	73.6	**<0.001**
	**distal**	I	2,27	2×10^{-4}	0.989	3.822	0.061	2,21	4.24	0.052	10.257	**0.004**	2,15	2.915	0.108	13.415	**0.002**
		S + I	2,26	1.157	0.33	7.054	**0.004**	2,20	6.367	**0.007**	9.725	**0.001**	2,14	17.71	**<0.001**	35.683	**<0.001**

Table S3. Results of comparisons between each major group via pANCOVA and ANCOVA, for the analysis of ΣF_{max}* v. m_{body}. Conventions as per Table S2.

anatomical region		test	mammals v. reptiles					mammals v. bipeds					reptiles v. bipeds				
			df	*F*	*P*	*F*†	*P*†	*df*	*F*	*P*	*F*†	*P*†	*df*	*F*	*P*	*F*†	*P*†
**forelimb**	**whole**	S	2,25	1.074	0.31	18.185	**<0.001**										
		I	2,25	4.428	**0.046**	32.703	**<0.001**										
		S + I	2,24	2.129	0.141	15.819	**<0.001**										
	**proximal**	S	2,26	0.596	0.447	12.827	**0.001**										
		I	2,26	2.498	0.126	18.567	**<0.001**										
		S + I	2,25	1.212	0.315	9.106	**0.001**										
	**distal**	S	2,27	1.909	0.178	21.094	**<0.001**										
		I	2,27	4.056	0.054	37.742	**<0.001**										
		S + I	2,26	2.066	0.147	18.26	**<0.001**										
**hindlimb**	**whole**	S	2,27	2.717	0.111	5.046	**0.033**	2,21	10.504	**0.004**	14.712	**0.001**	2,15	19.921	**0.001**	23.937	**<0.001**
		I	2,27	1.01	0.324	5.152	**0.031**	2,21	3.517	0.075	7.013	**0.015**	2,15	6.288	**0.024**	12.374	**0.003**
		S + I	2,26	1.406	0.263	3.1	0.062	2,20	5.004	**0.017**	7.102	**0.005**	2,14	9.645	**0.002**	12.757	**0.001**
	**proximal**	S	2,27	2.433	0.13	2.433	0.13	2,21	9.047	**0.007**	9.047	**0.007**	2,15	11.151	**0.005**	11.151	**0.005**
		I	2,27	1.331	0.259	1.331	0.259	2,21	4.302	0.051	4.302	**0.051**	2,15	4.82	**0.044**	4.82	**0.044**
		S + I	2,26	1.196	0.319	1.196	0.319	2,20	4.323	**0.028**	4.323	**0.028**	2,14	5.265	**0.02**	5.265	**0.02**
	**distal**	S	2,27	0.641	0.43	9.07	**0.006**	2,21	11.839	**0.003**	17.801	**<0.001**	2,15	28.666	**<0.001**	35.859	**<0.001**
		I	2,27	0.533	0.472	23.51	**<0.001**	2,21	4.122	0.055	8.827	**0.007**	2,15	6.412	**0.023**	38.294	**<0.001**
		S + I	2,26	0.486	0.621	11.588	**<0.001**	2,20	5.747	**0.011**	8.719	**0.002**	2,14	15.896	**<0.001**	36.533	**<0.001**

Table S4. Results of comparisons between each major group via pANCOVA, for the analysis of Σm_{muscle} v. m_{body} in the hindlimb. Here results are reported for analyses where the caudofemoralis longus was excluded from the reptile dataset (cf. Table 2). Conventions as per Table S2.

	test	mammals v. reptiles					mammals v. bipeds					reptiles v. bipeds				
		df	***F***	***P***	***F*†**	***P*†**	***df***	***F***	***P***	***F*†**	***P*†**	***df***	***F***	***P***	***F*†**	***P*†**
**whole (no CFL)**	S	2,42	1.11	0.298	28.181	<0.001	2,36	4.896	0.033	11.763	0.002	2,15	16.211	0.001	37.295	<0.001
	I	2,42	1.053	0.311	46.861	<0.001	2,36	2.605	0.115	10.143	0.003	2,15	12.464	0.003	53.946	<0.001
	S + I	2,41	0.928	0.404	28.051	<0.001	2,35	2.389	0.107	6.554	0.004	2,14	13.885	0.001	53.201	<0.001
**proximal (no CFL)**	S	2,32	2.84	0.102	34.385	<0.001	2,26	3.116	0.089	6.595	0.016	2,15	7.949	0.013	16.657	0.001
	I	2,32	2.118	0.155	43.572	<0.001	2,26	1.908	0.179	5.666	0.025	2,15	6.228	0.025	25.947	<0.001
	S + I	2,31	2.074	0.143	29.787	<0.001	2,25	1.507	0.241	3.564	0.044	2,14	5.274	0.02	15.599	<0.001

Table S5. Results of comparisons between each major group via pANCOVA and ANCOVA, for the analysis of mean F_{max}* v. m_{body} in the hindlimb. Here results are reported for analyses where the caudofemoralis longus was excluded from the reptile dataset (cf. Table 3). Other conventions as per Table S2.

	test	mammals v. reptiles					mammals v. bipeds					reptiles v. bipeds				
		df	***F***	***P***	***F*†**	***P*†**	***df***	***F***	***P***	***F*†**	***P*†**	***df***	***F***	***P***	***F*†**	***P*†**
**whole (no CFL)**	S	2,27	4.552	**0.042**	15.537	**0.001**	2,21	9.839	**0.005**	14.358	**0.001**	2,15	36.736	**<0.001**	70.841	**<0.001**
	I	2,27	0.438	0.514	6.135	**0.02**	2,21	3.198	0.088	6.785	**0.017**	2,15	5.568	**0.032**	13.676	**0.002**
	S + I	2,26	2.195	0.132	7.559	**0.003**	2,20	4.698	**0.021**	6.919	**0.005**	2,14	17.368	**<0.001**	34.604	**<0.001**
**proximal (no CFL)**	S	2,27	4.565	**0.042**	13.826	**0.001**	2,21	7.103	**0.015**	10.455	**0.004**	2,15	25.343	**<0.001**	46.338	**<0.001**
	I	2,27	0.654	0.426	5.392	**0.028**	2,21	3.053	0.095	5.882	**0.024**	2,15	6.279	**0.024**	12.562	**0.003**
	S + I	2,26	2.213	0.13	6.703	**0.005**	2,20	3.395	**0.054**	5.117	**0.016**	2,14	12.284	**0.001**	22.773	**<0.001**

Table S6. Results of comparisons between each major group via pANCOVA and ANCOVA, for the analysis of median F_{max}* v. m_{body} in the hindlimb. Here results are reported for analyses where the caudofemoralis longus was excluded from the reptile dataset (cf. Table S2). Other conventions as per Table S2.

	**test**	**mammals v. reptiles**					**mammals v. bipeds**					**reptiles v. bipeds**				
		df	***F***	***P***	***F*†**	***P*†**	***df***	***F***	***P***	***F*†**	***P*†**	***df***	***F***	***P***	***F*†**	***P*†**
	S	2,27	2.799	0.106	18.379	**<0.001**	2,21	7.608	**0.012**	14.396	**0.001**	2,15	28.975	**<0.001**	54.281	**<0.001**
**whole (no CFL)**	I	2,27	0.217	0.645	5.672	**0.025**	2,21	2.74	0.113	10.571	**0.004**	2,15	5.358	**0.035**	16.666	**0.001**
	S + I	2,26	1.356	0.275	8.85	**0.001**	2,20	3.647	**0.045**	7.763	**0.003**	2,14	14.461	**<0.001**	29.181	**<0.001**
	S	2,27	1.753	0.197	13.651	**0.001**	2,21	2.266	0.147	5.501	**0.029**	2,15	13.012	**0.003**	31.501	**<0.001**
**proximal (no CFL)**	I	2,27	0.296	0.591	4.764	**0.038**	2,21	1.232	0.28	6.399	**0.02**	2,15	6.176	**0.025**	17.244	**0.001**
	S + I	2,26	0.883	0.426	6.579	**0.005**	2,20	1.084	0.357	3.478	0.051	2,14	7.499	**0.006**	18.539	**<0.001**

Table S7. Results of comparisons between each major group via pANCOVA and ANCOVA, for the analysis of ΣF_{max}* v. m_{body} in the hindlimb. Here results are reported for analyses where the caudofemoralis longus was excluded from the reptile dataset (cf. Table S3). Other conventions as per Table S2.

	**test**	**mammals v. reptiles**					**mammals v. bipeds**					**reptiles v. bipeds**				
		df	***F***	***P***	***F*†**	***P*†**	***df***	***F***	***P***	***F*†**	***P*†**	***df***	***F***	***P***	***F*†**	***P*†**
	S	2,27	2.233	0.147	11.462	**0.002**	2,21	9.148	**0.006**	14.712	**0.001**	2,15	24.923	**<0.001**	39.397	**<0.001**
**whole (no CFL)**	I	2,27	1.346	0.256	19.545	**<0.001**	2,21	2.706	0.115	7.013	**0.015**	2,15	7.568	**0.015**	28.675	**<0.001**
	S + I	2,26	1.432	0.257	10.501	**0.001**	2,20	4.418	**0.026**	7.102	**0.005**	2,14	13.441	**0.001**	31.071	**<0.001**
	S	2,27	2.684	0.113	10.109	**0.004**	2,21	6.167	**0.022**	9.047	**0.007**	2,15	17.953	**0.001**	28.47	**<0.001**
**proximal (no CFL)**	I	2,27	2.02	0.167	14.135	**0.001**	2,21	2.071	0.165	4.302	**0.051**	2,15	7.858	**0.013**	19.375	**0.001**
	S + I	2,26	1.784	0.188	7.901	**0.002**	2,20	2.948	0.076	4.323	**0.028**	2,14	9.657	**0.002**	18.261	**<0.001**

Table S8. Results of comparisons between each major group via pANCOVA and ANCOVA, for the analysis of L^* v. m_{body} in the hindlimb. Here results are reported for analyses where the caudofemoralis longus was excluded from the reptile dataset (cf. Table 4). Other conventions as per Table S2.

	test	mammals v. reptiles					mammals v. bipeds					reptiles v. bipeds				
		df	*F*	*P*	*F*†	*P*†	*df*	*F*	*P*	*F*†	*P*†	*df*	*F*	*P*	*F*†	*P*†
**whole (no CFL)**	S	2,27	0.098	0.757	1.157	0.292	2,21	0.371	0.549	0.073	0.789	2,15	0.25	0.624	0.204	0.658
	I	2,27	0.001	0.974	0.305	0.585	2,21	0.624	0.438	1.437	0.244	2,15	0.681	0.422	1.375	0.259
	S + I	2,26	0.057	0.945	0.574	0.571	2,20	1.607	0.225	2.24	0.133	2,14	1.134	0.35	0.764	0.485
**proximal (no CFL)**	S	2,27	0.03	0.865	3.068	0.091	2,21	0.238	0.631	0.175	0.68	2,15	4×10^{-4}	0.985	0.731	0.406
	I	2,27	0.196	0.662	2.256	0.145	2,21	0.302	0.588	0.372	0.548	2,15	1.073	0.317	2.227	0.156
	S + I	2,26	0.094	0.91	1.616	0.218	2,20	0.828	0.452	0.966	0.398	2,14	0.805	0.467	1.061	0.372

Table S9. Results of comparisons between each major group via pANCOVA and ANCOVA, for the analysis of ΣPCSA v. Σm_{muscle} in the hindlimb. Here results are reported for analyses where the caudofemoralis longus was excluded from the reptile dataset (cf. Table 5). Other conventions as per Table S2.

	test	mammals v. reptiles					mammals v. bipeds					reptiles v. bipeds				
		df	*F*	*P*	*F*†	*P*†	*df*	*F*	*P*	*F*†	*P*†	*df*	*F*	*P*	*F*†	*P*†
**whole (no CFL)**	S	2,27	0.28	0.601	0.28	0.601	2,21	5.272	**0.032**	5.272	**0.032**	2,15	1.264	0.279	1.264	0.279
	I	2,27	1.5	0.231	1.5	0.231	2,21	0.225	0.64	0.225	0.64	2,15	0.658	0.43	0.658	0.43
	S + I	2,26	0.725	0.494	0.725	0.494	2,20	2.676	0.093	2.676	0.093	2,14	1.253	0.316	1.253	0.316
**proximal (no CFL)**	S	2,27	0.073	0.789	0.073	0.789	2,21	2.84	0.107	2.84	0.107	2,15	0.716	0.411	0.716	0.411
	I	2,27	0.075	0.787	0.075	0.787	2,21	0.313	0.582	0.313	0.582	2,15	0.095	0.763	0.095	0.763
	S + I	2,26	0.049	0.952	0.049	0.952	2,20	1.743	0.201	1.743	0.201	2,14	0.538	0.596	0.538	0.596

Table S10. Pan-amniote regression (pGLS) coefficients for each comparison, for hindlimb analyses where the caudofemoralis longus was excluded from the reptile dataset. These are reported for data on a $\log_{10}$ scale; also reported is the mean percent prediction error (%PE). Note that the coefficients are computed excluding bipeds, given that bipeds frequently differ from quadrupedal species.

comparison	slope under isometry	whole limb			proximal		
		**slope**	**intercept**	**mean %PE**	**slope**	**intercept**	**mean %PE**
Σm_{muscle} v. m_{body}	1.0	0.9812	-1.3788	63.91	1.0006	-1.4884	55.08
mean F_{max}* v. m_{body}	-0.333	-0.3116	0.1999	39.35	-0.3181	0.2262	40.22
median F_{max}* v. m_{body}	-0.333	-0.2926	0.0031	41.14	-0.2763	0.0369	46.03
ΣF_{max}* v. m_{body}	-0.333	-0.3106	1.6691	50.52	-0.3022	1.5098	45.2
L* v. m_{body}	0.333	0.2911	-1.5947	21.28	0.2988	-1.535	21.96
ΣPCSA v. Σm_{muscle}	0.666	0.7118	-1.8378	22.31	0.7122	-1.9303	21.52

4. Supplemental code

Included with the Supplemental Information is R computer code used to perform all analyses in the study, along with necessary input data files (muscle data in .csv format, phylogenetic tree in .nwk format). The code also allows for estimation of a particular variable via the pan-amniote regression given some user-supplied input (e.g., body mass estimate for an extinct species), along with 95% prediction intervals.
